# Shear wave elastography and Afirma™ gene expression classifier in thyroid nodules with indeterminate cytology: a comparison study

**DOI:** 10.1007/s12020-017-1509-9

**Published:** 2018-01-19

**Authors:** Ghobad Azizi, James M. Keller, Michelle L. Mayo, Kelé Piper, David Puett, Karly M. Earp, Carl D. Malchoff

**Affiliations:** 1 Wilmington Endocrinology, 1717 Shipyard Boulevard, Wilmington, NC 28403 USA; 2 Wilmington Pathology Associates, 1915 South 17th Street, Suite 100, Wilmington, NC 28401 USA; 3 Beth Israel Deaconess Medical Center, 109 Brookline, Suite 200, Boston, MA 02215 USA; 4 Carolina Arthritis, 1710 South 17th Street, Wilmington, NC 28401 USA; 5 UConn Health, 263 Farmington Avenue, Farmington, CT 06030 USA

**Keywords:** Shear wave elastography, Ultrasound, Thyroid nodule, Indeterminate FNAB, GEC testing

## Abstract

**Purpose:**

To compare shear wave elastography (SWE) and Afirma™ gene expression classifier (GEC) for diagnosis of malignancy in thyroid nodules (TNs) with Bethesda Classification (BC) III or IV indeterminate cytology.

**Methods:**

This preliminary single-center prospective study was approved by the Institutional Review Board. We evaluated 151 consented patients with 151 indeterminate TNs (123 BC III, 28 BC IV) on fine-needle aspiration biopsy (FNAB). B-mode ultrasound, vascularity, and SWE were performed prior to FNAB. TN stiffness was measured as shear wave velocity (SWV) in meters per second (m/s). The stiffest area of the TN was selected for SWV measurement. GEC testing was performed with a second FNAB. Surgery was recommended for GEC-suspicious TNs, or GEC-benign TNs with two or more worrisome B-mode US features.

**Results:**

Surgical pathology confirmed 31 malignant TNs. Among the GEC-suspicious group, 28 of 59 TNs were malignant. The SWV value of ≥3.59 m/s was the best cut-off for malignancy risk based on the receiver operating curve (ROC). Twenty-six malignant TNs had SWV ≥ 3.59 m/s. The sensitivity and specificity for SWV ≥ 3.59 m/s were 83.9 and 79.2%, respectively. Positive predictive value (PPV) was 51.0% and negative predictive value (NPV) was 95.0%. For the GEC-suspicious group, sensitivity, specificity, PPV, and NPV were 90.3, 74.2, 47.5, and 96.7%, respectively. In multivariate analysis, SWV and GEC-suspicious were significant predictors of malignancy, but B-mode features and vascularity were not.

**Conclusion:**

This preliminary study indicates that SWE and GEC are independent predictors of malignancy in TNs with BC III or IV.

## Introduction

Management of thyroid nodules (TNs) with indeterminate fine-needle aspiration biopsy (FNAB) has been a major challenge for thyroid experts. The Bethesda System for the Reporting of Thyroid Cytopathology renders standardized reporting criteria for TN FNAB cytopathology. Bethesda categories (BC) include: I (non-diagnostic or unsatisfactory), II (benign), III (atypia of undetermined significance or follicular lesion of undetermined significance), IV (follicular neoplasm or suspicious for follicular neoplasm), V (suspicious for malignancy) or VI (malignant) [1]. Indeterminate cytology categories include BC III and BC IV.

Approximately 15–30% of TN aspirations are interpreted as indeterminate, but the majority has benign surgical pathology. Among these patients, the risk for serious surgical complications ranges between 2–10% [2].

Prior to molecular testing, surgical resection was recommended for TNs with two indeterminate FNAB [1]. The risk stratification process has become more refined since molecular testing was introduced.

The use of molecular markers in indeterminate thyroid FNAB specimens improves diagnostic accuracy and subsequently influences the decision to perform surgery as well as the extent of the procedure. It is important to note that the long-term outcome data on the true value of this modality to guide therapeutic decision-making is lacking [3].

In the setting of indeterminate FNAB, some molecular tests have a high positive predictive value (PPV) in predicting malignancy [4], while others have a high negative predictive value (NPV) capable of predicting benign histology [2]. The combination of high NPV and sensitivity can be helpful to avoid surgery.

Several studies that used the Gene Expression Classifier (GEC) methodology for indeterminate TNs ≥ 10 mm reported a risk for thyroid cancer (TC) ranging from 13–47% [2, 5–7]. Practical limitations of molecular testing include cost and invasive nature of performing FNAB to obtain tissue samples for GEC or other molecular testing.

Elastography is a recent and non-invasive technology used to differentiate benign TNs from TC through tissue stiffness measurement [8]. Several recent publications report shear wave elastography (SWE) as an independent predictor of TC [9–12].

Virtual touch imaging quantification (VTIQ) is a 2D-SWE technology generated by acoustic radiation force impulse. VTIQ is capable of creating shear wave image and subsequent tissue quantification in one display. This allows for identification of regions for measurement of tissue stiffness [13]. The diagnostic performance of SWE in indeterminate TNs has not been evaluated in comparison to the GEC standard. We previously reported that TN stiffness measured by VTIQ-generated SWE is an independent predictor of TC when TNs with all Bethesda classifications were included. Based on the ROC curve, a single cut-off at 3.54 m/s has a sensitivity, specificity, PPV, and NPV of 79.27, 71.52, 26.75, and 96.34%, respectively [11]. Other predictors for TC were micro-calcifications and irregular margins.

The goal of this study was to prospectively compare the performance of SWE with GEC in TNs with indeterminate FNAB. Other B-mode ultrasound (US) characteristics and Color Doppler (CD) vascularity patterns were also examined and included in TC risk stratification.

## Materials and methods

### Study design

This single-center prospective study was approved by the Institutional Review Board. Patients were evaluated between April 2014 and October 2016. All participants gave written informed consent in this Health Insurance Portability and Accountability Act compliant study. All patients were examined clinically and with US prior to FNAB by a single practitioner with more than 15 years of experience in thyroid and neck US and 12 months experience using SWE.

### Study population and protocol

We prospectively evaluated 1520 patients with 1674 TNs from April 2014 to October 2016. All TNs were evaluated with a high-resolution US and FNAB. A total of 221 TN had BC III or IV; of those 160 had GEC testing. Fifty nine individuals with 61 TNs did not have GEC testing for a variety of reasons, including lack of insurance coverage for GEC testing or deciding for or against surgery without additional testing. This manuscript presents the data on TNs with GEC testing only.

Inclusion criteria included (a) age of 18 years and older; (b) TNs ≥ 10 mm or ≥5 mm with at least one suspicious US feature; (c) patients with indeterminate FNAB results (BC III or IV) and subsequent second FNAB with GEC methodology, and (d) surgical resection for those with GEC-suspicious results or GEC-benign and two or more suspicious B-mode US findings (irregular margins, microcalcification, central vascularity, tall shape, and hypoechoic pattern). All surgical candidates had cervical neck mapping and FNAB of lymph nodes, if indicated, prior to surgery. Nine patients were excluded because (a) they refused surgery after GEC-suspicious results (*n* = 5); (b) GEC testing was non-diagnostic (*n* = 2); initially four patients had non-diagnostic GEC testing, of these, two had successful second GEC and two refused repeat testing and were excluded; (c) when second FNAB yielded BC V or VI results, GEC was not performed (*n* = 2). Surgical pathology results were used as the reference standard for the classification of benign or malignant TN pathology.

### Conventional US examination and biochemical testing

Prior to FNAB, B-mode characteristics were examined with 18L6 High Definition probe. The initial US exam included B-mode information related to thyroid gland and TNs: homogenous versus heterogeneous gland, size of TN, sub-capsular location, isthmus location, macrocalcifications, microcalcifications, isoechoic, hypoechoic, hyperechoic, tall, solid, or complex TN. CD vascularity patterns of TNs were divided into four groups: no blood flow, peripheral blood flow only, peripheral and central blood flow and final group with primarily central blood flow. Sub-capsular location was assigned to lesions <2 mm from the thyroid capsule.

Serum TSH, thyroid peroxidase antibody (TPO Ab), thyroglobulin antibody (TgAb), thyroglobulin, and calcitonin were measured before FNAB as well. All measurements were performed by LabCorp (Research Triangle Park, NC).

### Shear wave elastography

SWE was the last part of the US exam prior to FNAB. For this study a Siemens Acuson S3000 US system was used. SWE was performed using VTIQ. During the elastography exam, patients were asked not to swallow or breathe for a few seconds. A qualitative elastography image was created first. Subsequently, SWV of the stiffest area within the TN was measured in meters per second (m/s) twice using a small region of interest (ROI) box measuring 1.5 mm in diameter (ROI box size with VTIQ is predetermined and cannot be changed). The highest velocity of Read 1 and Read 2 was reported as the maximum SWV, and the average of both reads was defined as mean velocity. SWV of the thyroid tissue surrounding the TN (tissue velocity) was measured once. SWV measurement can be converted from m/s to kilopascals (kPa) using the following formula: kPa (Young’s Modulus) = 3pc^2^, where *c* is shear wave speed in m/s and *p* is tissue density (a constant = 1000 kg/m^3^) [14]. For example, a SWV of 4 m/s is 48 kPa.

In addition to the main clinician of this study, three sonographers with 24, 7, and 14 years of experience, respectively, reviewed the SWE and B-mode features, including calcifications and irregular margins. They were blinded to the GEC result and surgical pathology outcomes. There was 100% agreement among all three reviewers for SWE image and SWV measurements. Among B-mode features, there was 96.9% agreement yielding a high inter-rater reliability, measured by Brennan and Prediger’s Kappa due to the skewed distribution of the ratings, of 93.8 [15].

### FNAB procedure, cytopathology, and GEC

FNAB was performed with US guidance to confirm accurate needle placement. For FNAB, 2–3 passes were made. Approximately six weeks after the first FNAB, a second FNAB was performed with two additional passes to obtain material for GEC analysis. Cytopathology was performed by Thyroid Cytopathology Partners (Austin, TX), who are associated with Veracyte. Afirma™ GEC testing was performed by Veracyte (South San Francisco, CA). Patients were enrolled only when GEC testing and repeat cytology with second FNAB was performed. 151 TNs met all inclusion criteria. 123 TNs were BC III, and 28 TN were BC IV.

### Statistical analysis

All statistical analyses were conducted using Stata 14.1 (StataCorp LP, College Station, TX, USA), and used a statistical significance level of 0.05 and two-sided hypothesis tests. Continuous variables were summarized using means and S.D., while categorical variables were summarized using frequencies and percentages. Bivariate associations with malignant pathology were assessed using Fisher’s exact tests for categorical variables and Wilcoxon rank-sum tests for continuous variables. Multivariate logistic regression was used to determine the influence of the maximum SWV and GEC on malignant pathology while controlling for all variables with a *p*-value of <0.1 in the bivariate analyses.

## Results

### Demographics

**Fig. 1:**
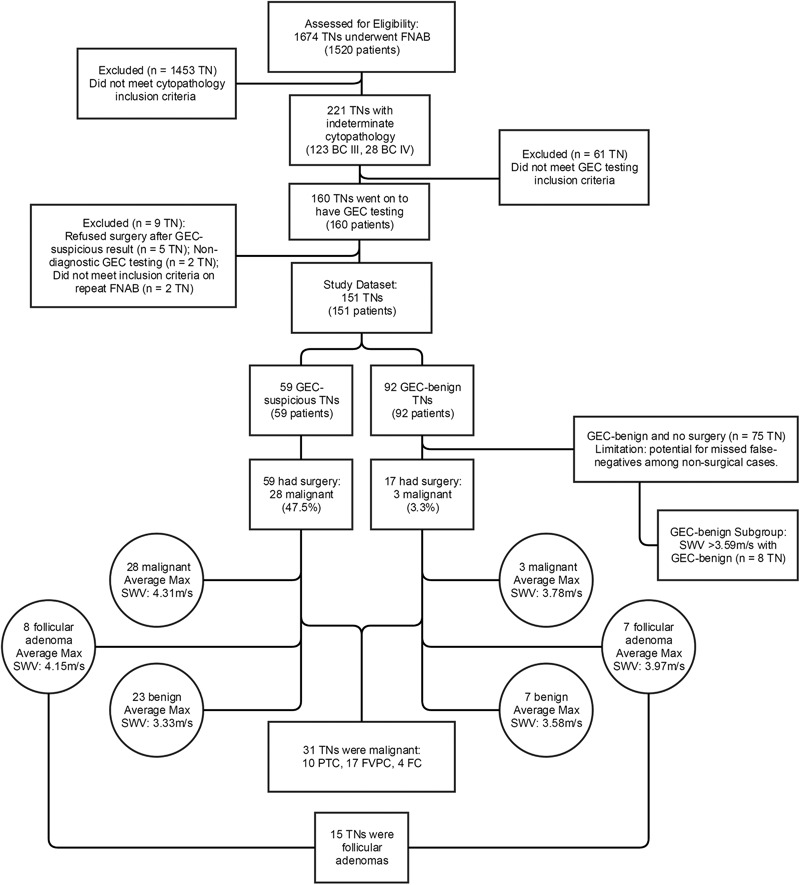
Flow chart of SWV & GEC results with surgical outcome

Figure 1 summarizes our study data. One hundred fifty one TNs in 151 mostly female patients (89.4%) met all inclusion criteria. Mean age was 51.4 years (S.D. 15.77) (Table 1). Neither age nor gender was a statistically significant predictor of TC.

**Table 1:** Demographic characteristics, US features, and clinical variables for the full study population as well as benign and malignant nodule pathology

	Full study population	Benign pathology^a^	Malignant pathology	*p*-value
	(*n* = 151)	(*n* = 120)	(*n* = 31)	
	*N* (%) or mean (S.D.)			
Female	135 (89.4%)	107 (79.3%)	28 (20.7%)	1.000
Age (years)	51.4 (15.77)	52.3 (16.00)	47.7 (14.49)	0.125
Bethesda classification^b^				
III	123 (81.5%)	101 (82.1%)	22 (17.9%)	0.119
IV	28 (18.5%)	19 (67.9%)	9 (32.1%)	
Heterogeneous gland	108 (71.5%)	83 (76.8%)	25 (23.2%)	0.267
Isthmus location	11 (7.3%)	7 (63.6%)	4 (36.4%)	0.238
Sub-capsular location	113 (74.8%)	88 (77.9%)	25 (22.1%)	0.491
Irregular margins	46 (30.5%)	33 (71.7%)	13 (28.3%)	0.130
Maximum nodule size (mm)	16.5 (11.96)	17.3 (12.39)	13.4 (9.63)	0.034
Single nodule gland	33 (21.9%)	22 (66.7%)	11 (33.3%)	0.051
MNG gland	118 (78.1%)	98 (83.0%)	20 (17.0%)	
Complex	17 (11.3%)	16 (94.1%)	1 (5.9%)	0.198
Solid	134 (88.7%)	104 (77.6%)	30 (22.4%)	
No calcifications	103 (68.2%)	88 (85.4%)	15 (14.6%)	0.007
Microcalcifications	31 (20.5%)	23 (74.2%)	8 (25.8%)	
Macrocalcifications	8 (5.3%)	3 (37.5%)	5 (62.5%)	
Microcalcifications and macrocalcifications	9 (6.0%)	6 (66.7%)	3 (33.3%)	
Tall	7 (4.6%)	6 (85.7%)	1 (14.3%)	1.000
Hypoechoic	50 (33.1%)	37 (74.0%)	13 (26.0%)	0.552
Isoechoic	99 (65.6%)	81 (81.8%)	18 (18.2%)	
Vascularity (Color Doppler)				0.349
No flow	41 (27.1%)	32 (78.1%)	9 (21.9%)	
Peripheral flow only	51 (33.8%)	37 (72.5%)	14 (27.5%)	
Peripheral > central flow	46 (30.5%)	40 (87.0%)	6 (13.0%)	
Central > peripheral flow	13 (8.6%)	11 (84.6%)	2 (15.4%)	
Elevated TPO Ab	56 (37.1%)	47 (83.9%)	9 (16.1%)	0.404
Elevated Tg Ab	45 (29.8%)	36 (80.0%)	9 (20.0%)	1.000
Elevated thyroglobulin	55 (36.4%)	43 (78.2%)	12 (21.8%)	0.835
Elevated calcitonin	3 (2.0%)	3 (100.0%)	0 (0.0%)	1.000
TSH	2.8 (5.74)	2.6 (4.11)	3.6 (9.87)	0.279
GEC-benign	92 (60.9%)	89 (96.7%)	3 (3.3%)	<0.001
GEC-suspicious	59 (39.1%)	31 (52.5%)	28 (47.5%)	

^a^This group includes all benign surgical results, as well as GEC-benign results without surgery. It presumes no additional false-negatives

^b^BC III: atypia or follicular lesion of undetermined significance and BC IV: follicular neoplasm or suspicious for follicular neoplasm

### Surgery

A total of 76 patients had thyroid surgery, including all 59 GEC-suspicious patients and all 17 with GEC-benign but two or more worrisome US features or worrisome TN size (>4 cm). There were 31 malignant TNs confirmed in 31 patients; ten were papillary thyroid cancer (PTC), 17 were follicular variant of PTC (FVPTC), and four were follicular carcinoma. One patient had metastatic tumor to lymph nodes. Among all surgical cases, 15 follicular adenomas (FAs) were detected, with eight in the GEC-suspicious group.

**Fig. 2:**
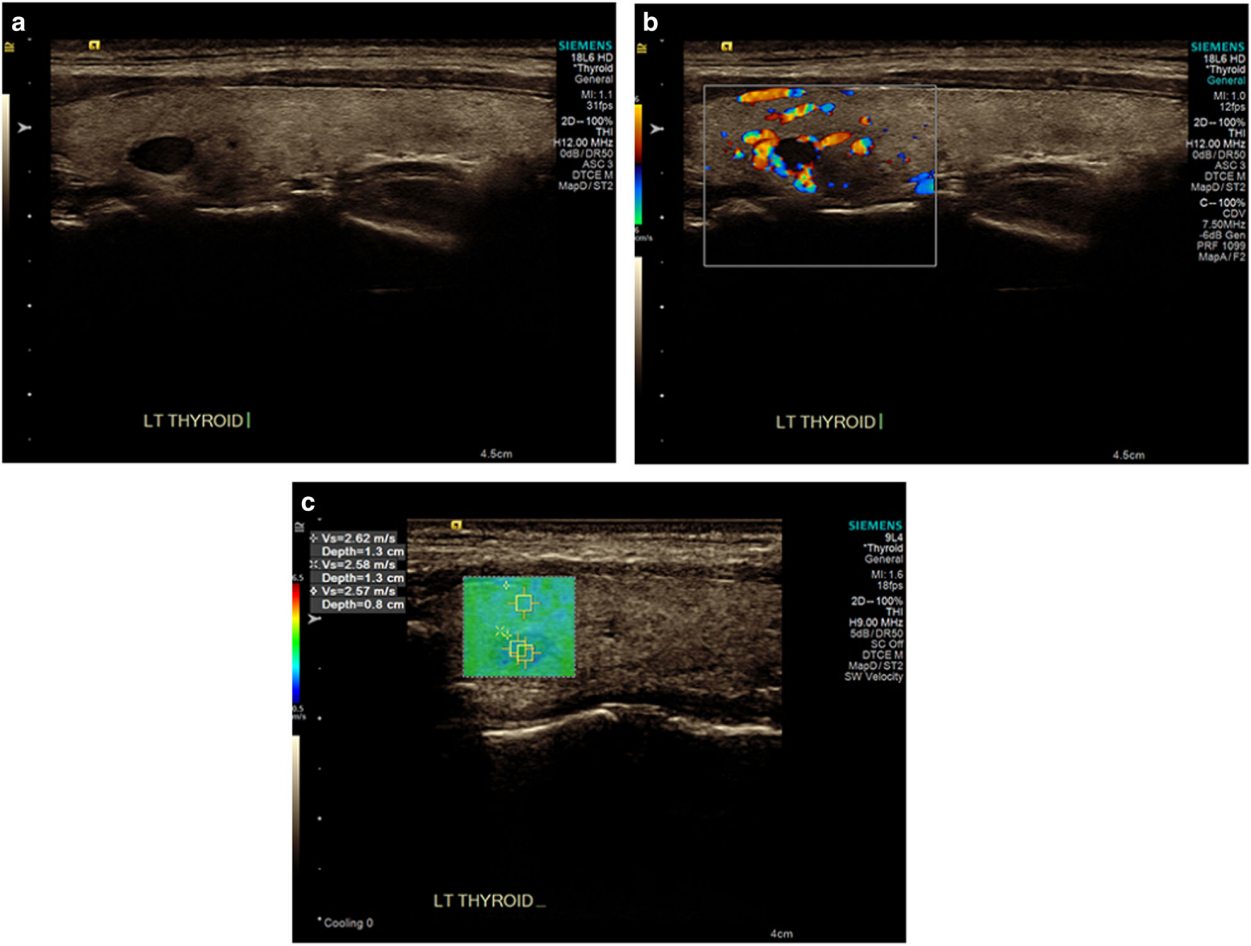
**a** An example of a B-mode image of a hypoechoic thyroid nodule in the left thyroid lobe measuring 7.2 × 4.6 × 4.4 mm. **b** Shows Color Doppler vascularity and **c** shows SWV measurements. Initial FNAB pathology was read as Atypia of Undetermined Significance (BC III); repeat FNAB confirmed this diagnosis and showed GEC-suspicious results. Surgical pathology showed a follicular adenoma

Figure 2 demonstrates B-mode, vascularity and elastography images of a TN with low SWV. FNAB pathology indicated BC III and GEC-suspicious results; final surgical pathology showed a FA.

### B-mode and Doppler analysis

B-mode US showed an average nodule size of 16.5 mm (S.D 11.96) (Table 1). Nodule size was, on average, significantly larger among benign pathologies with a mean of 17.3 mm (S.D. 12.39) than among malignant pathologies with a mean of 13.4 mm (S.D. 9.63) (*p* = 0.034). The presence of macrocalcifications was significantly higher in malignant TNs with 62.5% compared to 37.5% in benign TNs (*p* = 0.007). None of the other TN characteristics, however, showed a significant bivariate association with TC. Nodular blood flow on CD imaging was also not significantly different between the two groups.

### Serum analysis

None of the biochemical variables, including serum TSH, TPOAb, TgAb, thyroglobulin, and calcitonin, were significantly associated with TC (Table 1).

### GEC findings

**Fig. 3:**
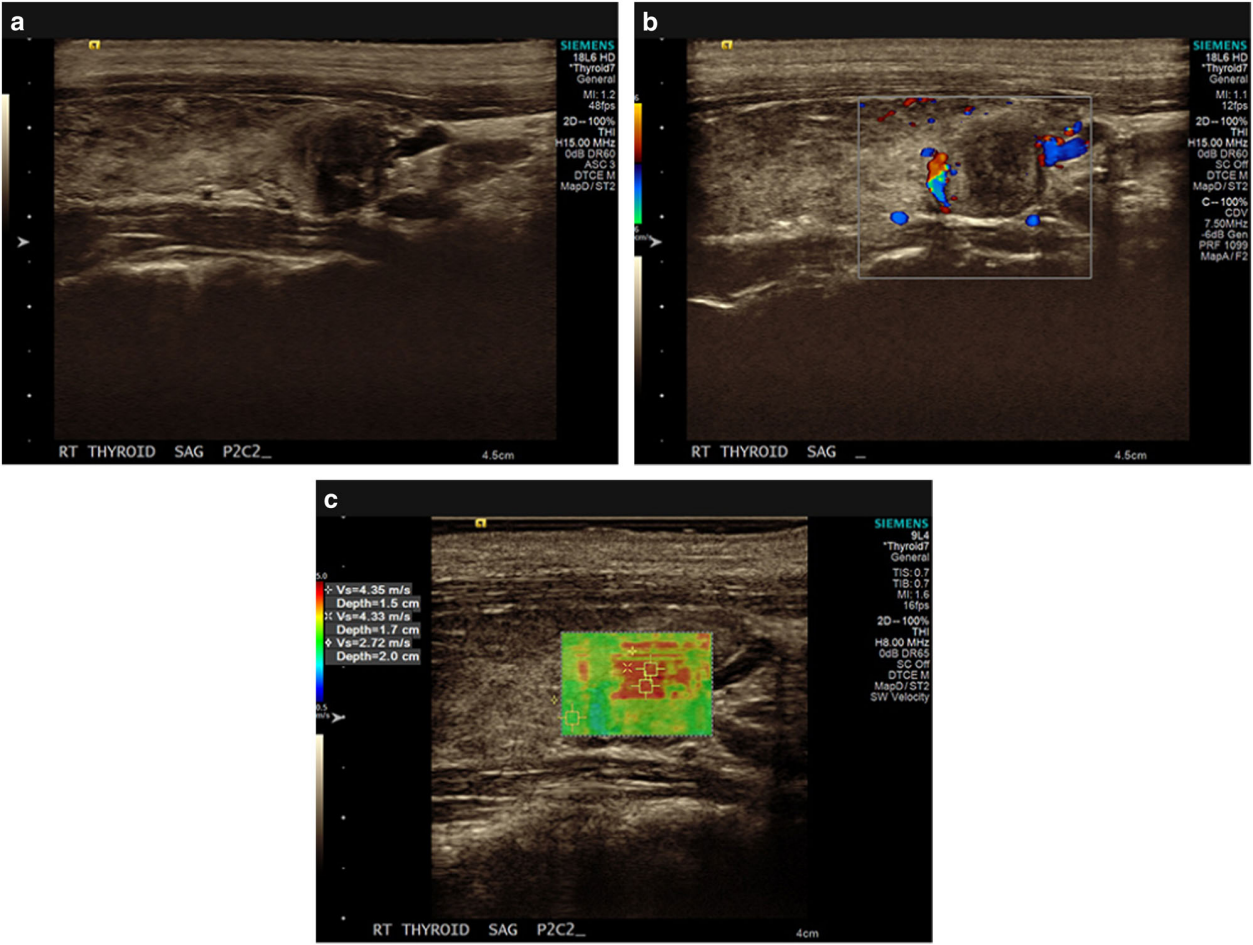
**a** Shows a B-mode image of a 12.1 × 11.4 × 9.1 mm thyroid nodule in the right lobe. **b** Shows Color Doppler vascularity and **c** shows SWV measurements. FNAB pathology showed Atypia of Undetermined Significance (BC III) and GEC-suspicious results. Surgical pathology confirmed a 10 × 8 × 5 mm FVPTC

GEC-benign results were found in 60.9% of TN. Among patients with GEC-suspicious results, 47.5% were found to be TC, and among those with GEC-benign, 3.3% were found to be TC (*p* < 0.001) (Table 1). The sensitivity and specificity of GEC as a predictor of TC were 90.3% and 74.2%, respectively, with a PPV of 47.5% and NPV of 96.7%, and a positive likelihood ratio (LR+) of 3.50 and a negative likelihood ratio (LR−) of 0.13 (Table 2). For nodules with both GEC-suspicious and a maximum SWV of ≥3.59 m/s as a predictor of TC (compared to either a GEC-benign, a maximum SWV of <3.59 m/s, or both) sensitivity was 77.4%, specificity was 91.7%, PPV was 70.6% and NPV was 94.0%, a LR+ was 9.29, and a LR− was 0.25. Figure 3 shows a TN with high SWV. FNAB pathology showed BC III and GEC-suspicious results. Surgical pathology was suggestive of un-encapsulated FVPTC.

**Table 2:** SWV sensitivity, specificity, positive and negative predictive values, and positive and negative likelihood ratios for predicting malignant nodule pathology for various cut-offs of maximum SWV and GEC-suspicious

Velocity max	Sensitivity (95% confidence interval)	Specificity (95% confidence interval)	Positive predictive value (95% confidence interval)	Negative predictive value (95% confidence interval)	Positive likelihood ratio	Negative likelihood ratio
≥3.59 (reference: <3.59)	26/31 = 83.9% (66.3–94.6%)	95/120 = 79.2% (70.8–86.0%)	26/51 = 51.0% (41.5–60.4%)	95/100 = 95.0% (89.5–97.7%)	4.03	0.20
≥4.0 (reference: <4.0)	16/31 = 51.6% (33.1–69.9%)	100/120 = 83.3% (75.4–89.5%)	16/36 = 44.4% (32.1–57.5%)	100/115 = 87.0% (82.1–90.6%)	3.10	0.58
≥4.5 (reference: <4.5)	8/31 = 25.8% (11.9–44.6%)	112/120 = 93.3% (87.3–97.1%)	8/16 = 50.0% (29.0–71.0%)	112/135 = 83.0% (79.7–85.8%)	3.87	0.79
GEC-suspicious	28/31 = 90.3% (74.3–98.0%)	89/120 = 74.2% (65.4–81.7%)	28/59 = 47.5% (39.5–55.5%)	89/92 = 96.7% (91.0–98.9%)	3.50	0.13
≥3.59 and GEC-suspicious (reference: either <3.59 or GEC-benign, or both)	24/31 = 77.4% (58.9–90.4%)	110/120 = 91.7% (85.2–95.9%)	24/34 = 70.6% (56.3–81.7%)	110/117 = 94.0% (89.1–96.8%)	9.29	0.25

### SWE findings

**Fig. 4:**
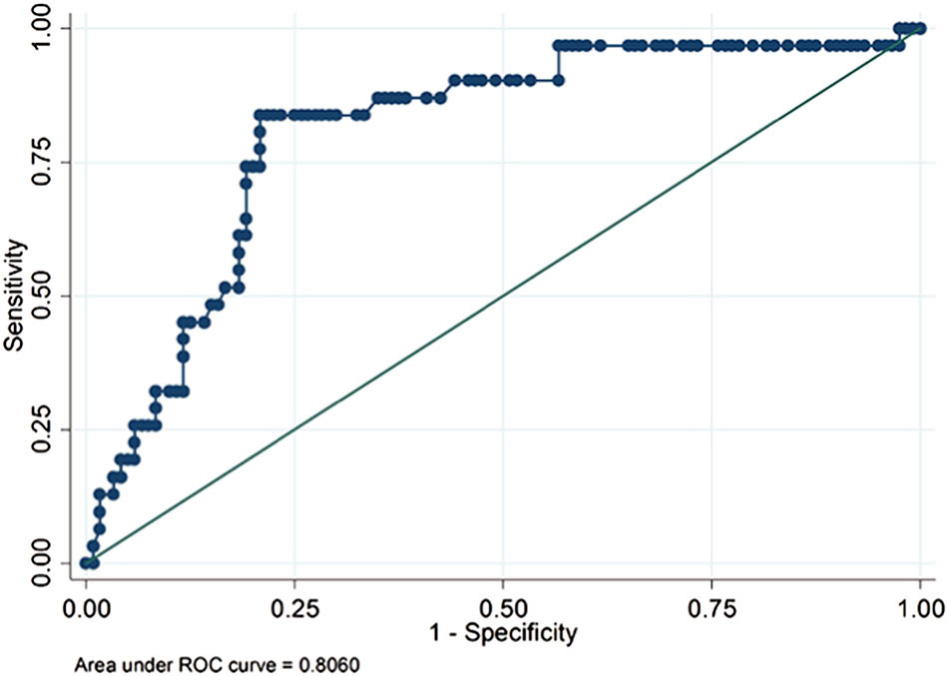
ROC curve using maximum nodule reading

SWV of the stiffest part of the nodule was measured twice and the readings were not significantly different from each other (*p* = 0.734, not in Table). Both the mean as well as the maximum of these two readings were significant predictors of TC (*p* < 0.001) (Table 3). The average maximum SWV in the benign group was 3.3 m/s (S.D. 0.96) compared to 4.3 m/s (S.D. 1.13) for the malignant group. The receiver operating curve (ROC) was used to determine the best single cut-off for maximum SWV to predict TN malignancy (Fig. 4). The area under the curve for this model is 0.8060 (95% CI: 0.72–0.89). A cut-off point of ≥3.59 m/s (38.66 kPa using Young’s Modulus conversion formula) had a sensitivity of 83.9%, a specificity of 79.2%, a PPV of 51.0% and a NPV of 95.0%, a LR+ of 4.03, and a LR− of 0.20 (Table 2). Table 2 also shows sensitivity, specificity, PPV and NPV, LR+ and LR− for other cut-off values. Our findings indicate that the higher the SWV, the greater the specificity, but lower the sensitivity.

**Table 3:** Shear wave elastography characteristics for the full study population as well as benign and malignant nodule pathology

	Full study population	Benign pathology^a^	Malignant pathology	*p*-value
	(*n* = 151)	(*n* = 120)	(*n* = 31)	
	Mean (S.D.)			
TN Velocity Read 1	3.4 (1.07)	3.2 (0.97)	4.2 (1.10)	<0.001
TN Velocity Read 2	3.3 (1.06)	3.1 (0.95)	4.1 (1.10)	<0.001
Maximum of Read 1 and Read 2	3.5 (1.07)	3.3 (0.96)	4.3 (1.13)	<0.001
Mean of Read 1 and Read 2	3.4 (1.06)	3.1 (0.95)	4.2 (1.08)	<0.001
Velocity tissue surrounding TN	2.8 (0.38)	2.8 (0.37)	2.9 (0.37)	0.192

^a^This group includes all benign surgical results, as well as GEC-benign results without surgery. It presumes no additional false-negatives

### Multivariate analysis

Among 59 GEC suspicious TNs, 28 were malignant. The median SWV for malignant TN in GEC-suspicious was 4.01 m/s (mean 4.31 m/s). For GEC suspicious TNs without TC on surgical pathology, the median SWV was 3.02 m/s (mean 3.54 m/s). This SWV is similar to that of the GEC-benign group.

All independent variables with a *p*-value < 0.1 in the bivariate analyses were included in a multivariate logistic regression model. In this context, calcifications, maximum nodule size and being a single nodule gland were not significant predictors of TC (Table 4). GEC-suspicious results and SWV of ≥3.59 m/s remained strong independent statistically significant predictors of TC (*p* < 0.001) with odds-ratios of 16.03 and 17.91, respectively.

**Table 4:** Multivariate logistic regression model predicting malignant nodule pathology including all predictors with a *p*-value of <0.1 in bivariate associations

Variable	Odds ratio	95% Lower confidence interval	95% Upper confidence interval	*p*-value
Maximum SWV ≥ 3.59 (reference: <3.59)	17.91	4.47	71.74	<0.001
GEC-suspicious (reference: GEC-benign)	16.03	3.80	67.56	<0.001
Microcalcifications	0.70	0.17	2.80	0.613
Macrocalcifications	4.52	0.25	80.24	0.304
Microcalcifications & macrocalcifications	0.11	0.00	3.73	0.223
(reference: none)				
Single nodule gland	3.26	0.87	12.24	0.080
(reference: MNG gland)				
Maximum nodule size (mm)	0.96	0.91	1.01	0.127

In an additional analysis, when we combined GEC-suspicious results with a SWV ≥ 3.59 m/s, we had a higher specificity and PPV than either criterion alone (Table 4). On the other hand, when a SWV cutoff of 2.9 m/s was used, among 51 TNs, 37 were GEC-benign and 13 were GEC-suspicious. We found only one TC.

Table 5 demonstrates an analysis of concordance between GEC and SWE groups with the purpose of better understanding our data.

**Table 5:** Table of concordance between SWE and GEC for TNs

	GEC result			
	GEC-benign^a^		GEC-suspicious	
Shear wave velocity	No cancer	Cancer	No cancer	Cancer
	(*n* = 89)	(*n* = 3)	(*n* = 31)	(*n* = 28)
<2.9 m/s	39	0	12	1
2.91–3.58 m/s	34	1	9	3
3.59–4.5 m/s	11	2	7	16
>4.5 m/s	5	0	3	8

^a^This group includes all benign surgical results, as well as GEC-benign results without surgery. It presumes no additional false-negatives

## Discussion

This single-center study prospectively determined SWV in TNs with indeterminate cytology (BC III or IV) plus a GEC analysis. SWV has a similar sensitivity, specificity, PPV, and NPV to that of GEC. High SWV (≥3.59 m/s) is a risk factor for TC. Our result for indeterminate TN with low SWV (<2.9 m/s) was also promising. Of 51 TNs (33.7%) with SWV of <2.9 m/s, only 1 TN was malignant. In this group, 39 were GEC-benign and 12 GEC-suspicious. SWV of ≥3.59 m/s and GEC-suspicious results are independent predictors of malignancy.

This may be particularly useful in subgroups of BC III and IV cytopathology. The first group is GEC-benign plus low SWV (2.9 m/s) that has a very low risk of TC. The second group is considered as high risk for TC (GEC-suspicious plus SWV ≥ 3.59). Combining both methodologies may serve us in new ways. While GEC testing can improve pre-operative risk stratification for TC, it cannot help with nodule selection. SWE may become a tool to assess TC risk prior to FNAB. It might not only improve nodule selection for FNAB, but also enhance the GEC interpretation. Adding SWV as an independent risk factor for TC can potentially increase the number of surgical procedures among GEC-benign nodules. It is important to mention that we do not suggest that surgery should be recommended solely based on high SWV (≥3.59 m/s). Future single and multi-center studies are needed to validate our results.

There are several differences in study design between this study and other studies using GEC methodology [2, 6, 7, 16–21]. In this study, all patients had SWE exam prior to FNAB. We included TN < 10 mm with at least one worrisome US feature. Furthermore, all patients in the GEC-suspicious group had a final surgical pathology diagnosis.

The frequency of final pathology diagnoses in this study was different than in other studies. In this study, the majority of TCs were determined to be FVPTC (55%). In most studies, all Bethesda classifications are included and the majority of TCs are PTC [16, 22–24]. TNs with indeterminate FNAB appear to have a different US profile when compared to TNs read as suspicious or diagnostic for TC (BC V and BC VI) on FNAB because the majority of the latter group are PTC [23–26]. In our current study, only one patient had metastatic lymph nodes. This is a lower prevalence of metastatic lymph nodes when compared to other publications [3, 27–31]. FVPTC may have a lower metastatic potential than PTC as reported in several other publications, including recent ATA guidelines [3, 27–31].

### Shear wave velocity (SWV) performance

A ROC analysis determined a single cut-off of SWV ≥ 3.59 m/s as the best predictor of TC with a sensitivity of 83.9%, a specificity of 79.2%, a PPV of 51.0%, and a NPV of 95.0%. This result is similar to our previous study with 707 TNs including all Bethesda categories [11].

We analyzed two additional cut-off values for SWV measurement (>4 m/s and >4.5 m/s). As anticipated, these had slightly higher specificity, but lower sensitivity (Table 2).

Among five malignant TNs with SWV < 3.59 m/s, three were <10 mm (2 FVPTC and 1 PTC). In this group, four were GEC-suspicious and one was GEC-benign.

We compared SWV with other B-mode US characteristics. By multivariate analysis B-mode US features were not predictors for malignancy in these indeterminate TNs. This confirms Samir’s findings [32]. This finding is contrary to our previous two elastography studies [11, 33]. Both studies reported that microcalcifications and irregular margins were independent risk factors for TC when all Bethesda categories were included. Excluding TNs with BC V and BC VI cytology on FNAB might be the main reason for this finding. The other reason is the smaller sample size of the current study.

### Comparison with other shear wave studies/technologies

Two studies with SuperSonic US system have evaluated the performance of SWE in nodules with indeterminate FNAB have yielded conflicting results.

Samir et al. demonstrates the potential benefit of SWV in TNs with atypical FNAB (BC III or BC IV) in a pilot study of 35 patients [32]. ROC determined a cut off value of 22.3 kPa, with sensitivity, specificity, PPV, and NPV were 82, 88, 75, and 91% respectively. His relatively low SWV is possible due to measurement of the entire TN versus a smaller area with highest SWV. In contrast, Bardet et al. examined BC III, BC IV, plus BC V TNs with SWE [34]. Of 131 patients enrolled, 21 (16%) had TC. The mean and maximum elastography measurements for benign and malignant nodules were similar. However, among malignant TNs, classic PTC had a higher SWV compared with other TC. In addition to using a different device, our procedure was different. Bardet et al. positioned the ROI box within the nodule in the area identified with elastography color map as most homogenous. In our study, we measured the stiffest area or highest SWV seen on color map.

Measurement of a larger area within a TN can decrease the overall SWV. For example, when using a larger ROI box 6 × 5 mm with Virtual Touch Quantification, the measured SWV is lower than when smaller ROI box of 1.5 × 1.5 mm of VTIQ is used [10, 11].

### GEC performance

Among 59 GEC-suspicious TNs, we found that 28 were malignant (47.5%). Our malignancy rate is similar to that of Alexander et al. [2, 17], Harrison et al. [21], and Yang et al. [18]. Our study confirmed that GEC has a relatively high NPV (96.7%), and suggests that there was no inherent selection bias in our study as compared to other studies in the literature.

In the GEC-benign group there were three malignant TNs, of those, two TNs had SWV ≥ 3.59 m/s. Two were FTC and one had PTC. The median SWV for the GEC-benign group was 3.00 m/s (mean 3.17 m/s). Among these patients, 17 had surgery with three malignancies. It is possible that additional nodules in this group may be malignant since not all TNs were resected. For example, eight TNs are being monitored despite SWV ≥3.59 m/s. This potential limitation of our study is a function of current clinical practice recommendations.

The malignancy rate for TNs < 10 mm was higher (26%) than TNs ≥ 10 mm (17.5%). A probable explanation is because smaller TNs (<10 mm) were selected for FNAB only when there was at least one worrisome US feature. There was no statistical difference in SWV between malignant TNs in both groups.

GEC testing for TNs ≥ 10 mm has been validated to describe the risk of thyroid malignancy in many studies [2, 6, 7, 16, 17, 19, 21]. Our analysis demonstrated that TN size did not affect GEC performance. This observation was reported by Wu et al. [20] as well.

### Autoimmune thyroid disease and TN with indeterminate FNAB

Some large studies demonstrated an association between differentiated TC and autoimmune thyroid disease and/or TSH when all Bethesda classifications were included [23–26]. There are at least three possible reasons that these relationships were not observed in this study. This relatively small study may have been underpowered to identify these associations. These associations may not occur in BC III and IV nodules. Alternatively, the final pathologies of this study were largely FVPTC and follicular carcinoma (69.2%), with only 30.7% being PTC. PTC may be more associated with autoimmune thyroid disease or abnormal TSH [22, 35].

### Follicular adenoma

Among the 15 FAs, eight were GEC-suspicious and nine had SWV ≥ 3.5 m/s. Based on our data, GEC and SWV have similar low specificity in distinguishing between FA and TC among TNs with indeterminate FNAB. However, this does not exclude the possibility of high sensitivity for TC. False positive GEC-suspicious results for FAs have been observed by others [2]. One possible reason for this, hypothesized by Nikiforov et al. is that FA may progress to follicular carcinoma [36].

### Limitations

Our study was a single center prospective study. Although all TNs in the GEC-suspicious group had surgery, not all in the GEC-benign group did. There are eight TNs in the GEC-benign group with high SWV (≥3.59 m/s) and ≤1 suspicious US feature. These individuals did not undergo surgical resection since GEC-benign result is felt to represent the current clinical practice standard [3]. We continue to monitor these eight TNs with US for growth and if indicated, repeat FNAB.

A number of factors can affect thyroid tissue stiffness and subsequently cause false SWV measurement: TNs with high fluid content (>50%), isthmus location, tissue fibrosis, and high calcified content of TNs (>25%).

There has been a recent nomenclature revision for encapsulated follicular variant of papillary thyroid cancer (EFVPTC). Due to its indolent behavior, this thyroid tumor was downgraded from cancer to noninvasive follicular thyroid neoplasm with papillary-like nuclear features (NIFTP) [36]. The implementation of this reclassification will lower the number of malignancies in particular among the GEC-suspicious group. Despite change in nomenclature, surgery is still the treatment of choice for this group of TNs. This recommendation was published in August 2016 and did not affect our study.

## Conclusion

In TNs with BC III and IV cytopathology, SWV, and GEC demonstrate similar diagnostic performance and are independent predictors of TC.
